# Lung ultrasound predicts clinical course but not outcome in COVID-19 ICU patients: a retrospective single-center analysis

**DOI:** 10.1186/s12871-021-01396-5

**Published:** 2021-06-28

**Authors:** Stephanie-Susanne Stecher, Sofia Anton, Alessia Fraccaroli, Jeremias Götschke, Hans Joachim Stemmler, Michaela Barnikel

**Affiliations:** 1 Medical Department II, LMU Hospital Munich, Marchioninistr. 15, 81377 Munich, Germany; 2 Medical Department III, LMU Hospital Munich, Marchioninistr. 15, 81377 Munich, Germany; 3 Medical Department V, LMU Hospital Munich, Marchioninistr. 15, 81377 Munich, Germany

**Keywords:** COVID-19, Lung ultrasound, Lung ultrasound score, Clinical course, Outcome, Deterioration

## Abstract

**Background:**

Point-of-care lung ultrasound (LU) is an established tool in the first assessment of patients with coronavirus disease (COVID-19). Purpose of this study was to evaluate the value of lung ultrasound in COVID-19 intensive care unit (ICU) patients in predicting clinical course and outcome.

**Methods:**

We analyzed lung ultrasound score (LUS) of all COVID-19 patients admitted from March 2020 to December 2020 to the Internal Intensive Care Unit, Ludwig-Maximilians-University (LMU) of Munich. LU was performed according to a standardized protocol at ICU admission and in case of clinical deterioration with the need for intubation. A normal lung scores 0 points, the worst LUS has 24 points. Patients were stratified in a low (0–12 points) and a high (13–24 points) lung ultrasound score group.

**Results:**

The study included 42 patients, 69% of them male. The most common comorbidities were hypertension (81%) and obesity (57%). The values of pH (7.42 ± 0.09 vs 7.35 ± 0.1; *p* = 0.047) and p_a_O_2_ (107 [80–130] vs 80 [66–93] mmHg; *p* = 0.034) were significantly reduced in patients of the high LUS group. Furthermore, the duration of ventilation (12.5 [8.3–25] vs 36.5 [9.8–70] days; *p* = 0.029) was significantly prolonged in this group. Patchy subpleural thickening (*n* = 38; 90.5%) and subpleural consolidations (*n* = 23; 54.8%) were present in most patients. Pleural effusion was rare (*n* = 4; 9.5%). The median total LUS was 11.9 ± 3.9 points. In case of clinical deterioration with the need for intubation, LUS worsened significantly compared to baseline LU. Twelve patients died during the ICU stay (29%). There was no difference in survival in both LUS groups (75% vs 66.7%, *p* = 0.559).

**Conclusions:**

LU can be a useful monitoring tool to predict clinical course but not outcome of COVID-19 ICU patients and can early recognize possible deteriorations.

**Supplementary Information:**

The online version contains supplementary material available at 10.1186/s12871-021-01396-5.

## Background

The infection with the severe acute respiratory syndrome coronavirus 2 (SARS-CoV2), COVID-19, leads to viral pneumonia and other organ manifestations like renal and liver failure, myocardial dysfunction, thrombotic complications, and neurologic illnesses [1]. The main cause of intensive care unit (ICU) admission remains lung failure. Older age, comorbidities, high sequential organ failure assessment (SOFA) score, lymphopenia, elevated troponin, and D-dimer have been reported to correlate with poor outcome [2].

In the last years, point-of-care ultrasound has increasingly been used to assess critically ill patients. Especially lung ultrasound (LU) is becoming more important. The sonographic signs e.g., B-lines, are useful in the diagnosis of acute respiratory failure or circulatory shock. Furthermore, LU can be used at the bedside of critically ill patients to assess the efficacy of ventilatory treatments and monitor the course of lung failure. It may also be used to detect and manage respiratory complications such as pneumothorax, atelectasis, and pleural effusions [3–5]. Several studies reported that LU findings correlate similar to high-resolution computed tomography (CT) findings with the clinical course of ICU patients treated for respiratory failure [6, 7].

During the corona pandemic, LU was extensively used in COVID-19 patients since the infection causes interstitial pneumonia [8, 9]. Manivel et al. developed a protocol (Coronavirus disease lung ultrasound in the emergency department protocol - CLUE protocol), which involves an anatomical parameter, the severity of lung changes, and a physiological parameter (oxygen requirement) to evaluate COVID-19 patients in the emergency room [10]. Lichter et al. showed that lung ultrasound score (LUS) at hospital admission strongly correlates with the need for invasive mechanical ventilation and is a strong predictor of mortality [11].

In this study, we analyzed whether LU performed at ICU admission can predict the clinical course and outcome of COVID-19 patients.

## Methods

### Study design and ethical approval

In this single-center retrospective study, we analyzed prospectively and systematically recorded data of lung ultrasound examinations. The study was approved by the ethics committee of the medical faculty of the Ludwig-Maximilians-University (LMU) Munich, IRB number 20–0227 and waived the need for informed consent because of the non-interventional design of the investigation.

### Patient selection and data collection

Between 30/03/2020 and 10/12/2020, we studied all consecutive adult patients with a positive polymerase chain reaction assay for SARS-CoV2 in a respiratory tract sample, admitted to the Internal Intensive Care Unit at LMU hospital Munich, Campus Großhadern. There were no exclusion criteria. Demographic data, comorbidities, medications, and laboratory findings were collected systematically. Baseline was defined as the day of ICU admission. At the beginning of the pandemic, we established a lung ultrasound protocol on our ICU for every COVID-19 patient admitted. LUS was recorded at admission, and in case of respiratory deterioration. All patients underwent a comprehensive LU in the first 6 hours after admission. Respiratory deterioration was defined as the need for intubation in spontaneously breathing patients, including patients treated with high-flow nasal cannula (HFNC) and/or non-invasive ventilation (NIV).

### Follow-up and outcomes

All medical records were daily reviewed to obtain clinical follow-up. Outcome analysis started at the time of the baseline LU exam. ICU length of stay (LOS), length of mechanical ventilation, and all-cause ICU mortality were the endpoints of the study.

### Lung ultrasound

LU was performed by the ICU physician on duty supervised by a senior physician with expertise in LU recording and interpretation with the same equipment (Venue, GE Healthcare). Each exam takes between 3 and 5 min (min) with the patient in a supine position. No change in position was needed for the exam.

**Fig. 1:**
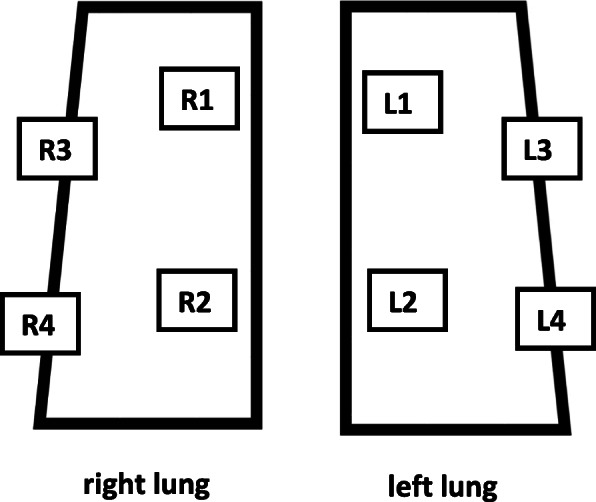
8 zones (R1-R4, L1-L4) for LU, adapted to CLUE protocol [10]

LU was performed on all COVID-19 patients admitted to the internal ICU using an eight-value method (four values for each lung) according to an adapted version of the CLUE protocol [10]. This protocol recommends scanning the chest systematically in 12 zones, six zones for the right lung (R1-R6) and six zones for the left lung (L1-L6). Due to the limited positioning options of our patients (mechanically ventilation, severe lung failure, hemodynamically unstable), we had to adapt the recently published CLUE-protocol. Instead of 12 we systematically scanned eight zones, we defined four zones for the right lung (R1 to R4), and four zones for the left lung (L1 to L4), see Fig. 1.

At each of the eight zones, LUS ranges from 0 to 3 points, with higher points allocated to severe lung changes (see Table 1). A normal lung will have a total score of 0 points. The worst LUS will be 24 points. All patients were divided into two groups depending on the LUS at admission: a low (0–12 points) and a high (13–24 points) LUS group.

**Table 1:** Lung ultrasound score according to CLUE protocol [10]

	LUS 0 points	LUS 1 point	LUS 2 points	LUS 3 points
A-lines	yes	no	no	no
B-lines	1–2	> 2	confluent	confluent
Pleural line	smooth, thin	irregular, thickened	irregular, thickened	irregular, thickened
Consolidation	no	no	yes, height < 1 cm	yes, height > 1 cm
Accessory				+/− air bronchogram +/− vascularity

### Statistical analyses

Continuous normally distributed data were presented as means ± standard deviation (SD) and compared using the Student’s t-test. Normal distribution was assessed by the Shapiro-Wilk test. Non-normally distributed variables were compared using the Mann-Whitney-U test. LU scores in consecutive exams were compared using the signed Wilcoxon signed-rank test. Correlation between data was examined using Pearson’s correlation coefficient or Chi^2^ test. *P*-values less than 0.05 were considered to indicate statistical significance. All data were analyzed with SPSS version 27 (IBM Corp., Armonk, NY).

## Results

During the study period, clinical data were collected for 42 consecutive COVID-19 patients admitted to the Internal ICU. Table 2 shows baseline characteristics and LU assessments of all patients, as well as grouped by LU severity. Twenty-four patients (57%) had a baseline LUS of 0–12 points, and 18 (43%) had a LUS of 13–24 points. The mean age was 66 ± 13 years and 69% were males. Comorbidities were present in 40 patients (95%) with hypertension (81%) being the most common followed by obesity (57%), diabetes (33%), and medication with immunosuppression (31%). Patients with high LUS suffered significantly more often from hematological malignancies (4.2 vs 27.8%, *p* = 0.033); in all cases lymphomas under ongoing chemotherapy plus anti-CD20-treatment. There were no differences in the laboratory findings, the SOFA or APACHE II scores for both LU groups. The values of pH (7.42 ± 0.09 vs 7.35 ± 0.1; *p* = 0.047) and p_a_O_2_ (107 [80–130] vs 80 [66–93] mmHg; *p* = 0.034) were significantly reduced in patients of the high LUS group. Furthermore, the duration of ventilation (12.5 [8.3–25] vs 36.5 [9.8–70] days; *p* = 0.029) was significantly prolonged in this group.

**Table 2:** Baseline characteristics

	All LUS (***n*** = 42)	LUS 0–12 (***n*** = 24)	LUS 13–24 (***n*** = 18)	***P***-value
**Characteristics at ICU admission**				
Age, years	66 ± 13	68 ± 12	64 ± 13	0.293
Male gender, n	29 (69)	17 (71)	12 (66.7)	0.775
SOFA	7.3 ± 3.7	7.1 ± 3.5	7.6 ± 4.1	0.656
APACHE II	19.8 ± 7.9	19.1 ± 7.6	20.7 ± 8.5	0.540
BMI, kg/m^2^	29.0 ± 4.8	28.7 ± 4.4	29.4 ± 5.3	0.649
**Medical history**				
Hypertension, n	34 (81)	20 (83.3)	14 (77.8)	0.654
Ischemic heart disease, n	9 (21.4)	5 (20.8)	4 (22.2)	0.915
Diabetes, n	14 (33.3)	7 (29.2)	7 (38.9)	0.513
Obesity, n	24 (57.1)	13 (54.2)	11 (61.1)	0.657
Solid tumor, n	3 (7.1)	3 (12.5)	0	0.124
Hematological malignancy, n	6 (14.3)	1 (4.2)	6 (27.8)	**0.033**
Immunosuppression, n	13 (31)	6 (25)	7 (38.9)	0.341
Solid-organ recipient, n	4 (9.5)	1 (4.2)	3 (16.7)	0.177
Transient ischemic attack/Stroke, n	5 (11.9)	4 (16.7)	1 (5.6)	0.277
Asthma, n	2 (4.8)	1 (4.2)	1 (5.6)	0.836
COPD, n	3 (7.1)	3 (12.5)	0	0.124
**Medications on ICU**				
ACE inhibitor, n	3 (7.1)	2 (8.3)	1 (5.6)	0.733
Angiotensin receptor blocker, n	9 (21.4)	6 (25)	3 (16.7)	0.52
Dexamethason, n	24 (57.1)	16 (66.7)	8 (44.4)	0.155
Other anti-inflammatories, n	26 (61.9)	17 (70.8)	9 (50)	0.174
**Baseline laboratory results**				
Leukocytes, G/l,	9.7 (5.6–11.3)	8.02 (5.6–12.7)	9.9 (6.7–11.2)	0.332
Lymphocytes, G/l	7 (3–10)	8.5 (5–10.3)	4.5 (2.8–8.3)	0.066
Creatinine, mg/dl	0.9 (0.7–1.75)	0.9 (0.73–1.1)	0.9 (0.6–2.5)	0.929
Blood urea nitrogen, mg/dl	50 (27–78)	47 (28–63)	58 (19–92)	0.751
Albumin, mg/dl	2.9 ± 0.5	3.0 ± 0.4	2.8 ± 0.5	0.066
C-reactive protein, mg/dl	10.3 (8.1–14.1)	10.1 (4.8–12.9)	10.6 (10–11.2)	0.083
Lactate dehydrogenase, U/l	412 ± 125	418 ± 128	405 ± 124	0.742
Interleukin-6, pg/ml	89.9 (40.3–197)	81.1 (40.3–239)	92 (39.8–167)	0.549
Ferritin, ng/ml	1533 (662–2201)	985 (322–2055)	1648 (1178–2571)	0.141
High sensitive Troponin T, ng/ml	0.018 (0.01–0.04)	0.014 (0.01–0.03)	0.022 (0.01–0.07)	0.187
D-Dimer, μg/ml	1.2 (0.68–4.25)	1.1 (0.6–1.88)	1.2 (0.78–6.83)	0.173
Lactate, mmol/l	1.1 (0.9–1.5)	1.1 (0.9–1.58)	1.0 (0.8–1.5)	0.351
Blood glucose, mg/dl	141 (118–179)	143 (117–193)	133 (117–177)	0.638
Brain natriuretic peptide, pg/ml	736 (212–1847)	677 (218–1725)	753 (193–8015)	0.688
**Respiration/Ventilation**				
Invasive ventilation at admission	19 (45.2)	9 (37.5)	10 (55.5)	0.250
p_a_O_2_/FiO_2_ ratio, mmHg	156 ± 66	167 ± 67	141 ± 64	0.213
pH	7.40 ± 0.10	7.42 ± 0.09	7.35 ± 0.1	**0.047**
p_a_O_2_, mmHg	91 (75–119)	107 (80–130)	80 (66–93)	**0.034**
p_a_CO_2_, mmHg	44.4 ± 16.6	41.8 ± 12.3	47.8 ± 19.2	0.249
Respiratory rate, breaths/min	24.3 ± 5.7	23.8 ± 6.2	24.8 ± 5.0	0.543
PEEP (IV), mbar	12.7 ± 4.1	7.8 ± 5.3	12.6 ± 3.8	0.409
Driving pressure (IV), mbar	12 (10–15)	11.5 (8.8–13.5)	12 (10–16)	0.393
Compliance (IV), ml/mbar	41.4 ± 12.8	44.9 ± 10.3	37.8 ± 14.9	0.254
Proning, n	12 (28.6)	6 (25)	6 (33.4)	0.559
ECMO, n	8 (19)	3 (12.5)	5 (27.8)	0.134
**Baseline lung ultrasound**				
Pleural effusion, n	4 (9.5)	2 (8.3)	2 (11.1)	0.764
Pleural thickening, n	38 (90.5)	20 (83.3)	18 (100)	0.072
Subpleural consolidations, n	23 (54.8)	7 (29.2)	16 (88.9)	**< 0.001**
Lung ultrasound score, n	11.9 ± 3.9	9.2 ± 2.4	15.7 ± 2.0	**< 0.001**
**Outcome**				
Mechanical ventilation, days	19 (9–51)	12.5 (8–25)	36.5 (10–70)	**0.029**
ICU LOS, days	15.5 (8–49)	15 (8–25)	38 (8–72)	0.203
ICU survival, n	30 (71.4)	18 (75)	12 (66.7)	0.559
Causes of death, n				
- Lung failure	3	2	1	
- Sepsis	4	2	2	
- Bleeding complication	4	2	2	
- Cardiac failure	1	0	1	

Data are given as median and interquartile range or n and percent or mean ± SD, respectively. *LUS* lung ultrasound score, *ICU* intensive care unit, *SOFA* sepsis-related organ failure score, *APACHE II* Acute Physiology And Chronic Health Evaluation II, *BMI* body mass index, *COPD* chronic obstructive lung disease, *ACE* angiotensin-converting enzyme, *IV* invasive ventilation, *p*_*a*_*O*_*2*_ partial pressure of oxygen, *FiO*_*2*_ fraction of inspired oxygen, *p*_*a*_*CO*_*2*_ partial pressure of carbon dioxide, *PEEP* positive end-expiratory pressure, *ECMO* extracorporeal membrane oxygenation, *LOS* length of stay

None of the patients had a normal LU at ICU admission or homogenous B-lines in all 8 zones. Patchy subpleural thickening (*n* = 38; 90.5%) and subpleural consolidations (*n* = 23; 54.8%) were present in most patients. Pleural effusion was rare (*n* = 4; 9.5%). The mean total LUS was 11.9 ± 3.9.

**Fig. 2:**
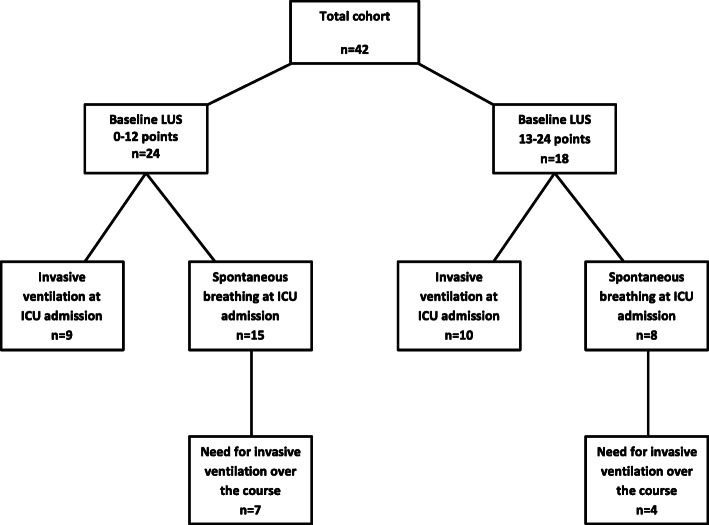
Course of invasive ventilation stratified by LUS groups

**Fig. 3:**
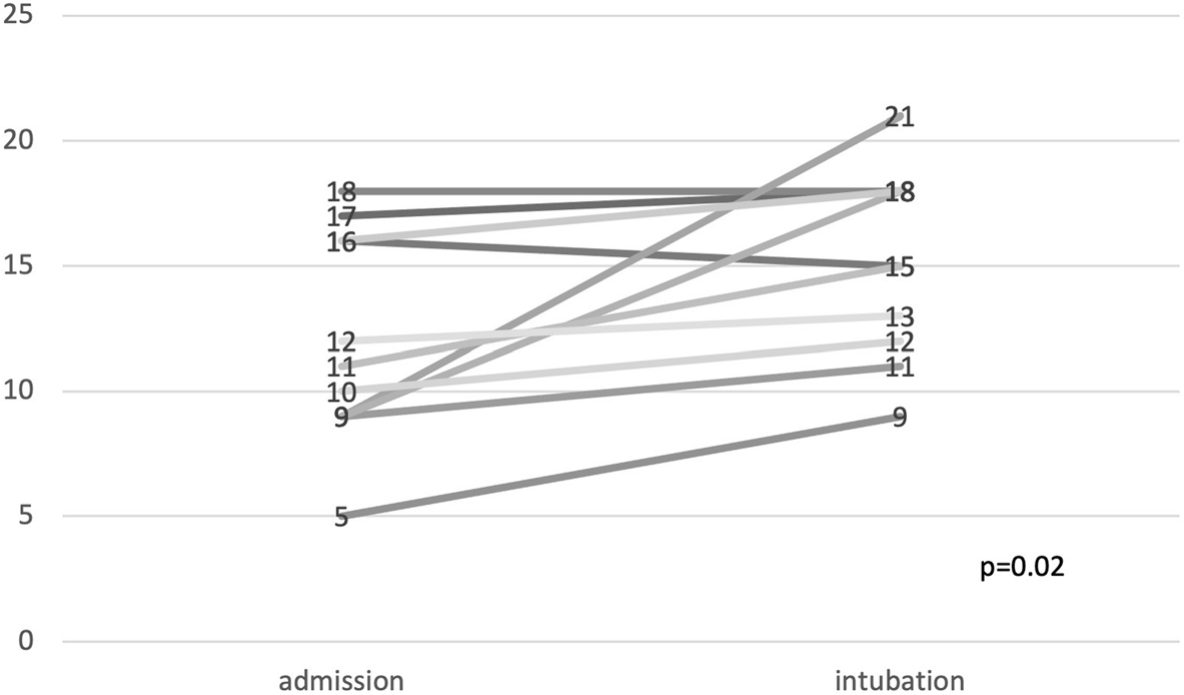
Course of LUS in patients with respiratory deterioration

Twenty-three patients (54.8%) were not mechanically ventilated at ICU admission. Eleven of whom worsened over the course (after 2 (1–7) days) with the need for intubation and invasive ventilation, see Fig. 2. In this patient group, LUS worsened mostly with increasing evidence of B-Lines, pleural thickening, and consolidations in the anterior zones, see also Table 3. The change in LUS from baseline (ICU admission) to clinical deterioration (day of intubation) was significant (*p* = 0.02), see Fig. 3.

**Table 3:** Lung ultrasound in respiratory deteriorating patients. Evidence of findings in at least one LU zone. Data are given as n and percent or mean ± SD

Parameter	Baseline (*n* = 11)	Follow-up (*n* = 11)	*P*-value
Pleural effusion	0 (0)	1 (9)	n.s.
Homogenous diffuse B-lines	0 (0)	0 (0)	n.s.
Pleural thickening	11 (100)	11 (100)	n.s.
Subpleural consolidations	5 (46)	10 (91)	**0.025**
Lung ultrasound score	12 ± 4.2	15.3 ± 3.7	**0.02**

In our cohort ICU mortality was 29% (*n* = 12). Baseline characteristics grouped by ICU mortality are shown in Supplemental Table 1. Non-survivors had a significantly increased SOFA (6.2 ± 3.4 vs 10 ± 3; *p* = 0.001) and APACHE II score (18 ± 7.6 vs 24 ± 7.1), *p* = 0.022). There was no difference in preexisting comorbidities in both groups. Non-survivors had significantly increased values of leucocytes (8.0 [5.5–10.3] vs 11.9 [9.3–15.4] G/l; *p* = 0.019), creatinine (0.9 [0.6–1.0] vs 2.0 [0.8–3.0] mg/dl; *p* = 0.047), lactate dehydrogenase (381 ± 116 vs 483 ± 119 U/l; *p* = 0.016), interleukin-6 (79 [18.6–171] vs 233 [73–280] pg/ml; *p* = 0.019), high sensitive troponin (0.012 [0.01–0.02] vs 0.053 [73–280] ng/ml, *p* < 0.001), and brain natriuretic peptide (482 [174–1454] vs 1725 [797–11,652] pg/ml; *p* = 0.023). Furthermore, lymphocytes (8 [5.0–10.8] vs 3 [2.3–7.3] G/l; *p* = 0.039), and albumin (3.0 ± 0.5 vs 2.6 ± 0.3 mg/dl; *p* = 0.008) were significantly decreased in non-survivors.

Values for p_a_O_2_/FiO_2_ ratio (171 ± 61 vs 118 ± 65, *p* = 0.017) and pH (7.42 ± 0.08vs 7.34 ± 0.13, *p* = 0.017) were significantly lower in the non-survivor group. Non-survivors needed proning (*p* = 0.008) and ECMO therapy (*p* < 0.001) significantly more often.

There was no difference in mortality between the LUS groups in our cohort. But presence of pleural effusion (*p* = 0.033) and subpleural consolidations (*p* = 0.020) were each significantly increased in the group that died.

## Discussion

COVID-19 primarily leads to viral pneumonia with all stages of lung failure beneath other organ manifestations [1]. During the COVID-19 pandemic, several hospitals used LU to determine the severity of lung failure and to support treatment decisions [8, 12]. In this retrospective study, we evaluated in 42 consecutive COVID-19 ICU patients the potential of LU to predict clinical course and outcome.

In our cohort, 24 patients were assigned to the low LUS group, and 18 patients to the high LUS group. Comparing the two groups there were no significant differences between age, sex, SOFA, APACHE II score, and all considered laboratory findings. This is surprising because several studies have shown that changes in these parameters are associated with higher severity of illness [13–15]. Other studies demonstrated that higher LU scores are associated with an increased disease severity [11, 16, 17]. The lack of relation might be explained by the differences in the studied cohorts. All considered studies examined patient cohorts of all hospital departments while our cohort consisted of ICU patients only.

As expected, pH and p_a_O_2_ were significantly reduced in the high LUS group confirming that LU reflects the severity of lung failure which is in line with the findings of Zhao et al. [18]. Furthermore, the duration of mechanical ventilation was significantly prolonged in the high LUS group whereas the length of ICU stay was not. The LOS is not only affected by the course of lung failure. Other organ failures like renal or liver, as well as circulatory failure prolong the LOS. In the opinion of the authors, these results show that LU score determined at ICU admission can predict the clinical course of lung failure in COVID-19 patients.

Twelve patients died during their ICU stay (29%). This mortality corresponds with the mortality predicted by SOFA (20–40%) [19] and APACHE II (25–40%) [20] score. There was no difference in mortality between the two LUS groups. One possible reason for these results is that only four of the 12 patients died because of lung failure. The main causes of death were bleeding complications (in all cases under heparinization because of ECMO) and sepsis with other focus than pneumonia. It seems to be comprehensible that LU cannot predict other causes of death than lung failure.

None of the COVID-19 patients in our study had a normal LUS at ICU admission. All of these patients were admitted to ICU due to respiratory failure with the need for oxygen supplementation. Therefore, it is plausible that no patient had a normal LU. Main findings of LU at admission were pleural thickening and subpleural consolidations, pleural effusions were rare, homogenous B-lines over all 8 zones were not seen. Interestingly, presence of pleural effusion and subpleural consolidations at baseline ultrasound examination were each significantly increased in the group that died. In particular, pleural effusions are not a typical feature of COVID-19 pneumonia but could be associated with other comorbidities like renal or heart failure that explain this result.

Other scientists have shown that laboratory parameters like increased values of C-reactive protein, interleukin-6, lactate dehydrogenase, and D-Dimer are predictors of mortality in COVID-19 [21–24]. In our cohort, levels of leukocytes, creatinine, albumin, lactate dehydrogenase, interleukin-6, high-sensitive troponin, and brain natriuretic peptide were significantly increased in the non-survivor group, while levels of lymphocytes and albumin were significantly lower.

In case of clinical deterioration with the need for intubation, LUS was significantly increased compared to initial LUS due to increased detection of B-lines, pleural thickening, and subpleural consolidations, especially in the anterior zones of the lung. These results show that LUS can be used as a valuable monitoring tool to assess the course of lung failure and can early recognize possible deteriorations which is in line with the findings of other research groups [11, 25].

### Limitations

This study has certain limitations. It is retrospective, based on data from a single-center and a small cohort. Therefore, a selection bias has to be presumed and data should be interpreted with caution. A larger number of subjects could potentially identify other characteristics associated with clinical course and outcome.

## Conclusion

In this retrospective study of 42 COVID-19 patients, we report that lung ultrasound score assessed at ICU admission can predict clinical course (increased days of invasive ventilation with higher lung ultrasound score) but not outcome. Lung ultrasound can be used as a valuable monitoring tool to assess the course of lung failure and can early recognize possible deteriorations.

## Supplementary Information

**Additional file 1: Supplemental Table 1.** Baseline characteristics adjusted to ICU death.

## Data Availability

The datasets used and/or analyzed during the current study are available from the corresponding author on reasonable request.

## Abbreviations

ACE: Angiotensin-converting enzyme
APACHE II: Acute Physiology And Chronic Health Evaluation II
BMI: Body mass index
COVID-19: Infection with the severe acute respiratory syndrome coronavirus 2
COPD: Chronic obstructive lung disease
CT: Computed tomography
ECMO: Extracorporeal membrane oxygenation
FiO2: Fraction of inspired oxygen
HFNC: High-flow nasal cannula
ICU: Intensive care unit
IV: Invasive ventilation
LMU: Ludwig-Maximilians-University
LOS: Length of stay
LU: Lung ultrasound
LUS: Lung ultrasound score
NIV: Non-invasive ventilation
paCO2: partial pressure of carbon dioxide
paO2: partial pressure of oxygen
PEEP: Positive end-expiratory pressure
SARS-CoV2: Severe acute respiratory syndrome coronavirus 2
SD: Standard deviation
SOFA: Sequential organ failure assessment
