# Supplementary material for: Lung ultrasound predicts clinical course but not outcome in COVID-19 ICU patients: a retrospective single-center analysis
Source: BMC Anesthesiol. 2021 Jun 28;21:178. doi: 10.1186/s12871-021-01396-5 (PMC8236568; doi:10.1186/s12871-021-01396-5)
Supplement: Supplementary file 1 — Additional file 1: Supplemental Table 1. Baseline characteristics adjusted to ICU death. [file 12871_2021_1396_MOESM1_ESM.docx]

| **Supplemental Table 1: Baseline characteristics adjusted to ICU death** | |  |  |
| --- | --- | --- | --- |
| \|  \| **All (n = 42)** \| **Survivors (n=30)** \| **Non-Survivors (n=12)** \| **P-value** \| \| --- \| --- \| --- \| --- \| --- \| \| **Characteristics at ICU admission** \| \| \| \| \| \| Age, years \| 66 ± 13 \| 64 ± 12 \| 72 ± 12 \| 0.065 \| \| Male gender, n \| 29 (69) \| 19 (63) \|  \| 0.211 \| \| SOFA \| 7.3 ± 3.7 \| 6.2 ± 3.4 \| 10 ± 3 \| **0.001** \| \| APACHE II \| 19.8 ± 7.9 \| 18 ± 7.6 \| 24 ± 7.1 \| **0.022** \| \| BMI, kg/m^2^ \| 29 ± 4.8 \| 29 ± 4.6 \| 28 ± 5.4 \| 0.596 \| \| **Medical history** \| \| \| \| \| \| Hypertension, n \| 34 (81) \| 23 (77) \| 11 (92) \| 0.269 \| \| Ischemic heart disease, n \| 9 (21.4) \| 6 (20) \| 3 (25) \| 0.724 \| \| Diabetes, n \| 14 (33.3) \| 9 (30) \| 5 (42) \| 0.474 \| \| Obesity, n \| 24 (57.1) \| 17 (57) \| 7 (58) \| 0.922 \| \| Solid tumor, n \| 3 (7.1) \| 2 (7) \| 1 (8) \| 0.852 \| \| Hematological malignancy, n \| 6 (14.3) \| 5 (17) \| 1 (8) \| 0.491 \| \| Immunosuppression, n \| 13 (31) \| 10 (33) \| 3 (25) \| 0.602 \| \| Solid-organ recipient, n \| 4 (9.5) \| 4 (13) \| 0 \| 0.189 \| \| Transient ischemic attack/Stroke, n \| 5 (11.9) \| 4 (13) \| 1 (8) \| 0.655 \| \| Asthma, n \| 2 (4.8) \| 2 (7) \| 0 \| 0.365 \| \| COPD, n \| 3 (7.1) \| 1 (3) \| 2 (17) \| 0.134 \| \| **Baseline laboratory results** \| \| \| \| \| \| Leukocytes, G/l, \| 9.7 (5.6-11.3) \| 8.0 (5.5-10.3) \| 11.9 (9.3-15.4) \| **0.019** \| \| Lymphocytes, G/l \| 7 (3-10) \| 8.0 (5.0-10.8) \| 3.0 (2.3-7.3) \| **0.039** \| \| Creatinine, mg/dl \| 0.9 (0.7-1.75) \| 0.9 (0.6-1.0) \| 2.0 (0.8-3.0) \| **0.047** \| \| Blood urea nitrogen, mg/dl \| 50 (27-78) \| 47 (27-60) \| 75 (32-146) \| 0.075 \| \| Albumin, mg/dl \| 2.9 ± 0.5 \| 3.0 ± 0.5 \| 2.6 ± 0.3 \| **0.008** \| \| C-reactive protein, mg/dl \| 10.3 (8.1-14.1) \| 10.2 (5.5-13.1) \| 12.1 (10.1-21.9) \| 0.068 \| \| Lactate dehydrogenase, U/l \| 412 ± 125 \| 381 ± 116 \| 483 ± 119 \| **0.016** \| \| Interleukin-6, pg/ml \| 89.9 (40.3-197) \| 79.4 (18.6-171) \| 233 (73-280) \| **0.019** \| \| Ferritin, ng/ml \| 1533 (662-2201) \| 1404 (468-1986) \| 2066 (865-2498) \| 0.387 \| \| High-sensitive Troponin T, ng/ml \| 0.018 (0.01-0.04) \| 0.012 (0.01-0.02) \| 0.053 (0.03-0.10) \| **<0.001** \| \| D-Dimer, µg/ml \| 1.2 (0.68-4.25) \| 1.1 (0.6-1.65) \| 4.5 (0.75-6.88) \| 0.072 \| \| Lactate, mmol/l \| 1.1 (0.9-1.5) \| 1.0 (0.8-1.2) \| 1.2 (1.0-1.6) \| 0.120 \| \| Blood glucose, mg/dl \| 141 (118-179) \| 144 (118-184) \| 129 (113-178) \| 0.597 \| \| Brain natriuretic peptide, pg/ml \| 736 (212-1847) \| 482 (174-1454) \| 1725 (797-11652) \| **0.023** \| \| **Respiration/Ventilation** \| \| \| \| \| \| IV at admission, n \| 19 (45.2) \| 10 (33) \| 9 (75) \| **0.015** \| \| P_a_O_2_/FiO_2_ ratio, mmHg \| 156 ± 66 \| 171 ± 61 \| 118 ± 65 \| **0.017** \| \| pH \| 7.40 ± 0.10 \| 7.42 ± 0.08 \| 7.34 ± 0.13 \| **0.019** \| \| p_a_O_2_, mmHg \| 91 (75-119) \| 91 (75-125) \| 86 (64-117) \| 0.504 \| \| p_a_CO_2_, mmHg \| 44.4 ± 16.6 \| 41 ± 14 \| 53 ± 20 \| **0.031** \| \| Respiratory rate, breaths/min \| 24.3 ± 5.7 \| 24.4 ± 6.2 \| 23.9 ± 4.3 \| 0.807 \| \| PEEP (IV), mbar \| 12.7 ± 4.1 \| 11.7 ± 3.7 \| 13.8 ± 4.4 \| 0.256 \| \| Proning, n \| 12 (28.6) \| 5 (17) \| 7 (58) \| **0.008** \| \| ECMO, n \| 8 (19) \| 2 (7) \| 6 (50) \| **<0.001** \| \| **Baseline lung ultrasound** \| \| \| \| \| \| Pleural effusion, n \| 4 (9.5) \| 1 (3) \| 3 (25) \| **0.033** \| \| Pleural thickening, n \| 38 (91) \| 27 (90) \| 11 (92) \| 0.870 \| \| Subpleural consolidations, n \| 23 (55) \| 13 (43) \| 10 (83) \| **0.020** \| \| Lung ultrasound score \| 11.9 ± 3.9 \| 11.4 ± 3.4 \| 13.3 ± 5 \| 0.154 \| \| **Outcome** \| \| \| \| \| \| Mechanical ventilation, days \| 19 (9-51) \| 33 (11-56) \| 11 (5-26) \| 0.138 \| \| ICU LOS, days \| 16 (8-49) \| 18 (8-49) \| 16 (10-46) \| 0.650 \| |  |  |  |
| Data are given as median and interquartile range or n and percent or mean ± SD, respectively. LUS: lung ultrasound score; ICU: intensive care unit; SOFA: sepsis-related organ failure score; APACHE II: Acute Physiology And Chronic Health Evaluation II; BMI: body mass index; COPD: chronic obstructive lung disease; IV: invasive ventilation; p_a_O_2_: partial pressure of oxygen; FiO2: fraction of inspired oxygen*;* p_a_CO_2_: partial pressure of carbon dioxide; PEEP: positive end-expiratory pressure; ECMO: extracorporeal membrane oxygenation; LOS: length of stay. |  |  |  |
